# Gene Regulation in Comorbid Migraine and Myogenic Temporomandibular Disorder Pain

**DOI:** 10.3390/genes16121435

**Published:** 2025-12-01

**Authors:** Ran Tao, Sufang Liu, Hui Maltezos, Feng Tao

**Affiliations:** Department of Biomedical Sciences, Texas A&M University College of Dentistry, Dallas, TX 75246, USA; rantao@tamu.edu (R.T.); shuhui@outlook.com (H.M.)

**Keywords:** migraine headache, temporomandibular joint pain, comorbidity, gen regulation, ferroptosis

## Abstract

Background/Objectives: Previous studies have demonstrated an association between migraine headache and temporomandibular joint disorders (TMDs), with a higher prevalence of TMD symptoms in patients with migraine. Methods: In this study, we conducted RNA sequencing to identify differentially expressed genes (DEGs) in the spinal trigeminal nucleus caudalis of mice with migraine-like pain and/or myogenic TMD. Results: We observed 204 upregulated and 274 downregulated genes in the comorbid migraine and TMD group compared to the control group. We identified 15 ferroptosis-related DEGs enriched in the pathways of neurodegeneration, cellular homeostasis, interleukin signaling, and pain response. Gene Ontology analysis highlighted the involvement of neuroinflammatory response and monoamine transmembrane transporter activity, while Gene Set Enrichment analysis showed enrichment in chemokine signaling, cell cycle, and calcium signaling pathways. Immune infiltration analysis identified M0 macrophages, immature dendritic cells, neutrophils, and eosinophils as key responders. Hub genes in the protein–protein interaction network included *Gm7536*, *Rpl17*, *Rpl22l1*, *Rpl14*, *Rps8*, *Rps29*, *Rpl35*, *Gm4889*, *Gm11808*, *Rps27rt*, *Rps12-ps3*, *Rpl10-ps3*, *Gm9843*, *Oas1c*, *Il1b*, and *Serpine1*, indicating their synergistic roles in such orofacial pain comorbidity. Conclusions: Our results suggest that the comorbid migraine and TMD can regulate gene expressions involving ferroptosis and immune cell responses and the identified DEGs could be targeted to develop novel therapies for this painful comorbidity.

## 1. Introduction

Migraine is a neurological disorder characterized by recurrent moderate to severe headaches [1]. Temporomandibular joint disorders (TMDs) are painful conditions affecting the jaw joint and muscles controlling jaw movement, including masticatory muscle disorder, disk displacements, and joint-based disorders [2,3]. myofascial TMD is often considered the dominant subtype, with its prevalence reported in some cohorts as high as 45.3% [4]. An association between migraine headache and TMDs has been shown in previous studies [5,6,7,8,9]. A higher prevalence of TMD symptoms is observed in migraineurs compared to the general population [10,11]. However, the underlying mechanisms for this association remain to be investigated.

Peripheral and central sensitization mechanisms are believed to underlie both migraine and TMDs. For instance, central sensitization has been shown to contributes to cutaneous allodynia in migraine and promote progression to chronic migraine [12]. On the other hand, TMD pain may involve amplification within peripheral and central nociceptive pathways [13,14].

Certain chronic pain disorders often coexist, with women being more susceptible than men [15]. Comorbid pain conditions encompass a variety of commonly co-occurring disorders, including TMDs, migraine, chronic headache, which is collectively referred to as chronic overlapping pain conditions (COPCs) [16]. It has been demonstrated that TMDs are associated with other pain conditions including migraine headache [17,18]. Giving the higher prevalence of chronic overlapping pain conditions in women, we conducted RNA sequencing (RNA-seq) in the present study to specifically identify altered gene expression in female mice, thereby providing insights into female-specific mechanisms for the comorbidity of TMDs and migraine.

Bulk RNA-seq is a high-throughput sequencing technique used to analyze the expression levels of all genes in mouse tissues, which has broad applications in pain research. This technique can help researchers understand the molecular mechanisms of pain and identify potential therapeutic targets [19,20,21]. In this study, we performed bioinformatics analysis on the dataset from the RNA-seq to explore novel mechanisms that underlie the association between migraine and TMDs. By examining gene expression patterns, we identified differentially expressed genes (DEGs) in the spinal trigeminal nucleus caudalis (Sp5C) of mice with migraine-like pain and/or myogenic TMD. Specifically, we analyzed DEGs associated with ferroptosis in the comorbidity condition.

In this study, our primary objective is to identify DEGs in comorbid migraine and myogenic TMD, and our secondary objective is to explore enriched pathways and molecular targets that could underlie this comorbidity condition.

## 2. Materials and Methods

### 2.1. Animals

Twelve eight-week-old female C57BL/6 mice (The Jackson Laboratory, Bar Harbor, ME, USA) were used in this study, housed under standard conditions with a 12 h light/dark cycle, with access to food and water ad libitum. All procedures were conducted under our animal use protocol (#2022-0159) approved by the Texas A&M University Institutional Animal Care and Use Committee. Animal handling and experiments adhered to the NIH Guide for the Care and Use of Laboratory Animals. Sample sizes were calculated using the power analysis program G*Power 3.1 [22].

### 2.2. Comorbid Migraine and TMD Mouse Model

Unilateral masseter muscle tendon ligation (TL) was performed to produce myogenic TMD. Mice were anesthetized with pentobarbital sodium (50 mg/kg, i.p.). A 3 mm incision was made on the left intraoral site, the tendon of the anterior masseter muscle was freed and tied with two 6.0 chromic gut ligatures 2 mm apart. The incision was closed with Vetbond tissue adhesive. Sham controls underwent the same procedure without tendon ligation. On day 8 after TL, the mice received a single intraperitoneal injection of nitroglycerin (NTG, 1 mg/kg). For the combined model, NTG was injected on day 8 post-TL. The mice in the control group received NTG dissolvent (30% ethanol, 30% propylene glycol, 40% double-distilled water) diluted in saline [5,6].

### 2.3. RNA-Seq and Bioinformatics Analysis

In general, Mice were randomly assigned to Sham, NTG, TL, and the combined TL_NTG groups. Sp5C tissues were collected one day after NTG injection. RNA quality was ensured before library preparation, followed by RNA sequencing on the Illumina HiSeq platform (Novogene Corporation Inc., Sacramento, CA, USA). Differential gene expression analysis was conducted using the DESeq2 package (version 1.36.0) with a cutoff of log2FC > 0.8 and adjusted *p*-value (padj) < 0.05. Heatmaps were generated with the Pheatmap package (version 1.0.13).

### 2.4. DEGs Associated with Ferroptosis

In this study, ferroptosis-related genes were collected from multiple sources. Firstly, GeneCards (https://www.genecards.org/ (accessed on 28 May 2023)) was used to search for genes associated with ferroptosis, resulting in 442 ferroptosis-related genes. Secondly, 388 ferroptosis-related metabolism genes were obtained from the FerrDb database (http://www.zhounan.org/fer-rdb/legacy/index.html (accessed on 28 May 2023)). After merging and removing duplicates, a total of 684 ferroptosis-related genes were identified (Table S1).

### 2.5. Enrichment and Immune Infiltration Analysis

In this study, Gene Ontology (GO) enrichment analysis was performed to explore the biological processes, molecular functions, and cellular components associated with significantly enriched DEGs. All ferroptosis-related genes were searched in the Metascape database (https://metascape.org/gp/index.html#/main/step1 (accessed on 28 May 2023)), and the ClueGO plugin in Cytoscape (v3.9.2) was used for enrichment analysis. Kyoto Encyclopedia of Genes and Genomes (KEGG) pathway and Gene Ontology (GO) terms enriched among the DEGs were identified using a hypergeometric overrepresentation test. The resulting *p*-values were adjusted for multiple comparisons using the false discovery rate (FDR) approach, and GO terms with FDR ≤ 0.05 were considered significant Additionally, gene set enrichment analysis (GSEA) was conducted using the GSEA software (version 4.3.3). To assess the levels of immune cell infiltration in the samples, immune infiltration was analyzed using CIBERSORT (https://cibersort.stanford.edu/ (accessed on 28 May 2023)).

### 2.6. Generation of Protein–Protein Interaction (PPI) Network

A PPI network was constructed to identify the biological interactions among DEGs. The String database was utilized to determine the genes and interactions, which were designated as nodes and lines, respectively. The resulting network was visualized using Cytoscape software. To identify hub genes, the cytoHubba plug-in was employed, which utilized Maximum Neighborhood Component, Stress, Degree, Closeness, and Radiality calculation methods. Additionally, the Cytoscape MCODE plug-in was used to extract core sub-networks from the PPI network.

### 2.7. Construction of Ferroptosis-Related DEGs and Other DEGs Co-Expression Network

The co-expression network was built to reveal interactions between ferroptosis-related DEGs and other DEGs. We computed Pearson correlation coefficients between all gene pairs. Gene pairs with correlation ≥0.95 were retained. The network was visualized using OmicStudio (https://www.omicstudio.cn/tool (accessed on 28 May 2023)). In the resulting graph, each gene is a node, and the nodes are connected by edges to visualize the interactions between genes.

## 3. Results

### 3.1. Identification of DEGs Associated with Ferroptosis in the Comorbid Orofacial Pain

**Figure 1:**
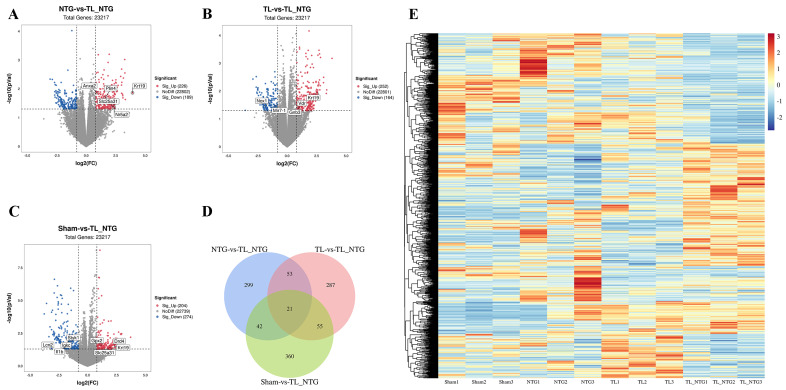
Identification of DEGs associated with ferroptosis in the comorbid migraine and myogenic TMD. (**A**–**C**) The NTG, TL, or Sham group was compared with the combined TL-NTG group to identify DEGs. There are 226 upregulated genes and 189 downregulated genes in the NTG vs. the TL_NTG group (**A**). There are 252 upregulated genes and 164 downregulated genes in the TL vs. the TL_NTG group (**B**). There are 204 upregulated genes and 274 downregulated genes in the Sham vs. the TL_NTG group (**C**). (**D**) The DEGs in the three comparisons were mapped and 21 DEGs were identified at the same time. (**E**) Heatmaps of the DEGs were created with pheatmap package (version 1.0.13).

Ferroptosis is a regulated form of cell death characterized by the accumulation of lipid-based reactive oxygen species (ROS), and its orchestration involves a complex interplay of specific genes. There are 684 distinct ferroptosis-associated genes in GeneCards and FerrDb databases. In this study, we compared gene expressions in the Sham, NTG, or TL group with the combined TL_NTG group to identify DEGs. DEGs based on mRNA expression were presented as a volcano map using the ggplot2 package (Version 3.4.2). There are 226 upregulated genes and 189 downregulated genes in the NTG vs. the TL_NTG group (Figure 1A). There are 252 upregulated genes and 164 downregulated genes in the TL vs. the TL_NTG group (Figure 1B). There are 204 upregulated genes and 274 downregulated genes in the Sham vs. the TL_NTG group (Figure 1C). The DEGs in the three comparisons were mapped (Figure 1D), and 21 DEGs were identified at the same time, including *Sncg*, *Slc5a7*, *Dbh*, *Chodl*, *Slc6a2*, *Slc18a3*, *Zfp185*, *Lhx4*, *Ntrk1*, *Crym*, *Phospho1*, *Vsig8*, *Gm6565*, *Fam166a*, *Gm13936*, *Krt19*, *Slfn4*, *6030498E09Rik*, *Gm11966*, *Gm5620*, and *G6pc*. Heatmaps of the DEGs were created with pheatmap package (version 1.0.13) (Figure 1E). By mapping with ferroptosis-related genes, we identified 15 ferroptosis-related DEGs: *Anxa2*, *Lcn2*, *Igkc*, *Slc25a31*, *Vdr*, *Krt19*, *Il1b*, *Plin4*, *Nr5a2*, *Styk1*, *Mir7-1*, *Nox1*, *Gpx2*, *Gnb3*, and *Drd4*. To assess whether the observed overlap between ferroptosis-related genes and disease-associated DEGs was greater than expected by chance, we performed a two-sided Fisher’s exact test using all quantified genes as the background universe. The test yielded *p* = 0.0667 with an odds ratio (OR) of 0.43, indicating that the overlap did not reach statistical significance. Ferroptosis-related DEGs were mapped to potential drug–gene interactions using the Drug-Gene Interaction Database (Figure S1). We also showed DEG analysis results for NTG vs. Sham, TL vs. Sham, and TL-NTG vs. Sham in Supplementary Materials (Tables S1–S3). The pairwise overlap analysis revealed 74 shared DEGs between NTG vs. Sham and TL vs. Sham, 132 shared DEGs between TL vs. Sham and TL-NTG vs. Sham, and 77 shared DEGs between NTG vs. Sham and TL-NTG vs. Sham. A total of 39 DEGs were common in all three comparisons, including *Fen1*, *Gzmk*, *Jmjd7*, *Idi1-ps1*, *Kansl2-ps*, *Pgk1-rs7*, *Styk1*, *Tmsb15b2*, *Tpt1-ps3*, and *Ube2n-ps1*.

### 3.2. Immune Infiltration in the Comorbid Orofacial Pain

**Figure 2:**
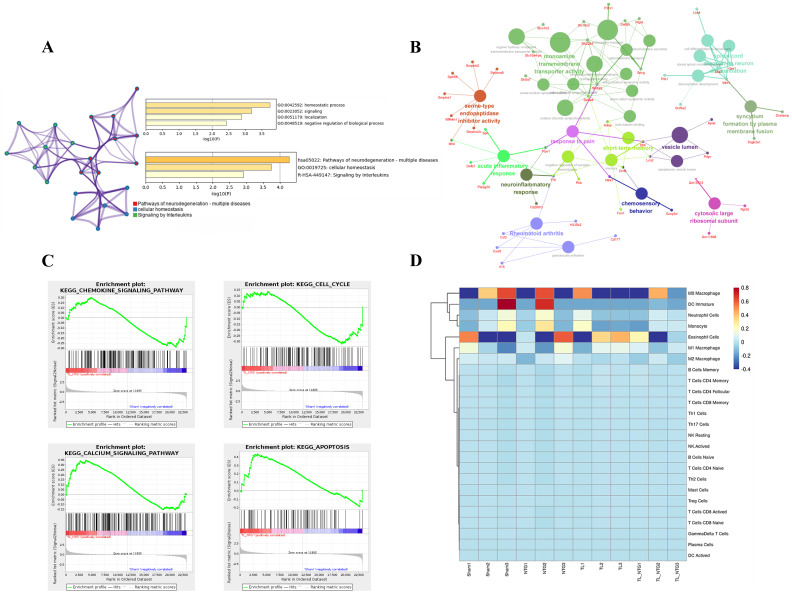
Immune infiltration in the comorbid migraine and myogenic TMD. (**A**) KEGG analysis showed that the ferroptosis-related DEGs are primarily enriched in the pathways associated with neurodegeneration, cellular homeostasis, and interleukin signaling. (**B**) GSEA indicated that these DEGs were significantly involved in chemokine signaling, cell cycle regulation, and calcium signaling pathways. (**C**) GO analysis identified multiple biological pathways, including response to pain, neuroinflammatory response, and monoamine transmembrane transporter activity, with *Lcn2*, *Il1b*, and *Trpv1* as key representative genes. (**D**) Immune infiltration analysis showed that the primarily involved immune cells were M0 macrophages, immature dendritic cells, neutrophils, and eosinophils.

KEGG analysis revealed that the ferroptosis-related DEGs are primarily enriched in the pathways associated with neurodegeneration, cellular homeostasis, and interleukin signaling (Figure 2A). GSEA indicated that these DEGs were significantly involved in chemokine signaling, cell cycle regulation, and calcium signaling pathways (Figure 2B). GO analysis identified multiple biological pathways, including response to pain, neuroinflammatory response, and monoamine transmembrane transporter activity, with *Lcn2*, *Il1b*, and *Trpv1* as key representative genes (Figure 2C). Given the close link between the enrichment analysis results and immune responses, we conducted an immune infiltration analysis. This analysis showed that the primarily involved immune cells were M0 macrophages, immature dendritic cells, neutrophils, and eosinophils (Figure 2D).

### 3.3. PPI Network in the Comorbid Orofacial Pain

**Figure 3:**
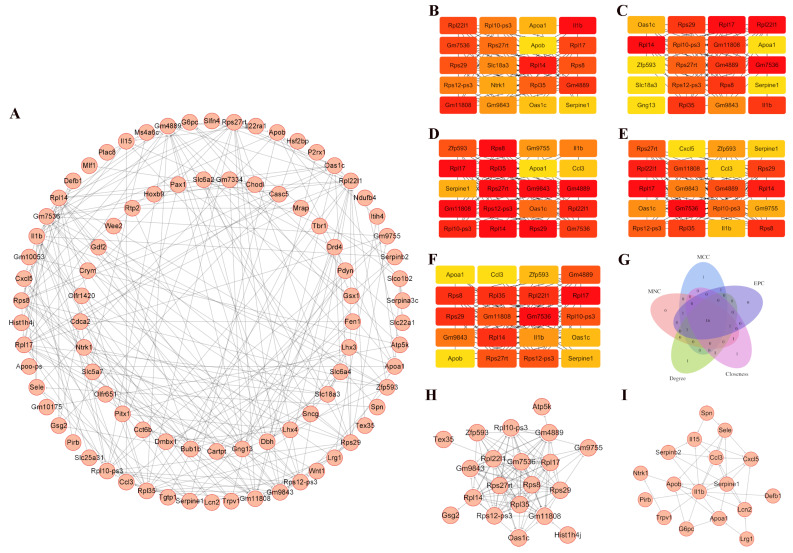
PPI network in the comorbid migraine and myogenic TMD. (**A**) Total PPI network was generated using Cytoscape software and the STRING database. (**B**–**F**) The top 20 hub genes were determined by maximal clique centrality (**B**), maximum neighborhood component (**C**), edge percolated component (**D**), degree (**E**), and closeness algorithms (**F**). (**G**) The whole hub genes included *Gm7536*, *Rpl17*, *Rpl22l1*, *Rpl14*, *Rps8*, *Rps29*, *Rpl35*, *Gm4889*, *Gm11808*, *Rps27rt*, *Rps12-ps3*, *Rpl10-ps3*, *Gm9843*, *Oas1c*, *Il1b*, and *Serpine1*. (**H**,**I**). The interactive density region in the PPI network by “MCODE’’ plug-in was discovered, which showed the top two dense regions in the total network.

The protein–protein interaction (PPI) network for the DEGs was constructed via STRING and visualized in Cytoscape (Figure 3A). The top 20 hub genes were determined using the cytoHubba plug-in, which utilized the maximal clique centrality, maximum neighborhood component, edge percolated component, degree, and closeness algorithms (Figure 3B–F). The whole hub genes calculated were mapped together (Figure 3G). These hub genes included *Gm7536*, *Rpl17*, *Rpl22l1*, *Rpl14*, *Rps8*, *Rps29*, *Rpl35*, *Gm4889*, *Gm11808*, *Rps27rt*, *Rps12-ps3*, *Rpl10-ps3*, *Gm9843*, *Oas1c*, *Il1b*, and *Serpine1*. The interactive density region in the PPI network by “MCODE’’ plug-in was also discovered, highlighting key interactions among these hub genes (Figure 3H–I). For instance, *Gm11808* binds to ribosomal proteins, such as, Rps12-ps3, Rps29, Rps8, while *Il1b* binds to Trpv1, Ccl3, Apob, Apoa1 and Lcn2. The expression patterns of the identified *Il1b*-interacting genes from the MCODE-enriched subnetwork were validated using the Allen In Situ Hybridization (ISH) Database (Figure S2).

### 3.4. Co-Expression Networks Between Ferroptosis-Related DEGs and Other DEGs

**Figure 4:**
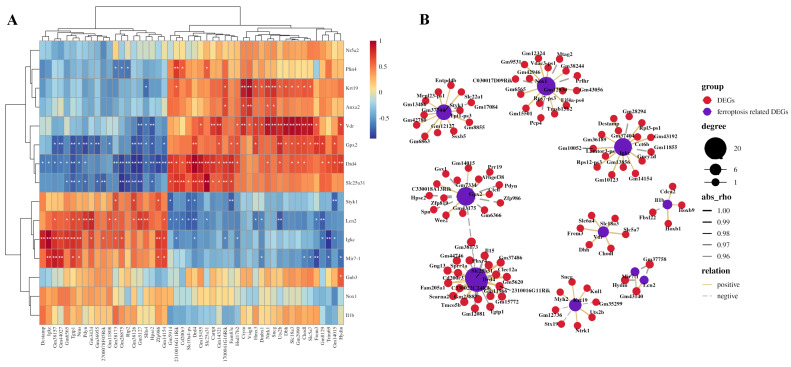
Co-expression networks between ferroptosis-related DEGs and other DEGs. (**A**) A gene co-expression network was established based on the correlation between the differential expression levels of the ferroptosis-related DEGs and other DEGs. Pearson correlation coefficient (PCC) was calculated, and R-value was used to determine the correlation coefficient, with a cutoff of PCC ≥ 0.95. The top 50 pairs of co-expression relationships were shown. * *p* < 0.05; ** *p* < 0.01. (**B**) The co-expression network between ferroptosis-related genes and DEGs was visualized. For example, *Gpx2* was found to co-express with *Pdyn*, *Ctvfl*, *Zfp819*, *Spn*, *Hpse2*, *Wee2*, and *Gm14015*, while *Ded4* and *Slc23a31* co-expressed with *Phxr4*, *Sprr1a*, *Tgtp1*, and *Tmco5b*.

A gene co-expression network was established based on the correlation between the differential expression levels of the ferroptosis-related DEGs and other DEGs. Pearson correlation coefficient (PCC) was calculated, and R-value was used to determine the correlation coefficient, with a cutoff of PCC ≥ 0.95. The top 50 pairs of co-expression relationships were shown in Figure 4A, and the co-expression network between ferroptosis-related genes and DEGs was visualized using OmicStudio tools and presented in Figure 4B. For example, *Gpx2* was found to co-express with *Pdyn*, *Ctvfl*, *Zfp819*, *Spn*, *Hpse2*, *Wee2*, and *Gm14015*, while *Ded4* and *Slc23a31* co-expressed with *Phxr4*, *Sprr1a*, *Tgtp1*, and *Tmco5b*. In addition, *Nox1* was found to co-express with *Rps7-ps3* and *Pcp4*.

## 4. Discussion

The comorbidity of migraine and TMDs arises from several overlapping mechanisms, including shared pain pathways as the trigeminal nerve branches converge at the spinal trigeminal nuclei [23,24]. Peripheral and central sensitization due to inflammation and persistent pain input increase pain sensitivity in both conditions [14,25]. Myofascial trigger points in masticatory muscles can provoke migraine headache, while cross-excitation among trigeminal nerve branches allows pain in one branch to trigger pain in another [26]. Elevated levels of calcitonin gene-related peptide in TMDs can lead to migraine, highlighting its role in neurogenic inflammation [27]. Epidemiological data show that migraine is about 2–3 times more common in women than in men, with hormonal fluctuations often exacerbating attacks (e.g., during the menstrual cycle) [28]. Similarly, in patients with TMDs, women exhibit a significantly higher prevalence—some studies report female: male ratios of 2–4:1 [29]. In our previous studies, we observed that a low dose of NTG combined with masseter muscle tendon ligation caused comorbid migraine and TMD pain, which lasted longer in female mice compared with male mice [5,6]. This sex-specific result is consistent with clinical epidemiological report and suggests that female susceptibility exists in the comorbidity condition. Additionally, increased dynorphin expression in the Sp5C, specifically in females, is linked to overlapping pain in TMDs and migraine [6]. Beyond the Sp5C, other central nervous system regions also play important roles in central sensitization relevant to migraine and TMD comorbidity. In migraine, the trigeminocervical complex, thalamus, brainstem nuclei (such as the periaqueductal gray and dorsolateral pons), and cortical areas modulating descending pain control are often implicated [12]. In TMD pain, structural and functional alterations have been reported in the somatosensory cortex, prefrontal cortex, and basal ganglia, suggesting that maladaptive neuroplasticity in these brain structures contributes to chronic TMD pain [30].

Ferroptosis, a regulated form of cell death marked by iron-dependent lipid peroxidation, has distinct biochemical features and regulatory mechanisms [31]. It has been implicated in various neurological and inflammatory disorders [32]. It has been demonstrated that ferroptosis contributes to neuroinflammation and oxidative stress, which are common features of orofacial pain like migraine and TMDs [33]. The lipid peroxidation and ROS generated during ferroptosis can trigger neuroinflammation, a key mechanism for chronic pain [34]. Additionally, cell membrane damage and neuronal death caused by ferroptosis can lead to nervous system dysfunction and cause pain induction. Inflammatory mediators and ROS produced during ferroptosis can decrease the pain threshold and enhance sensitivity to pain stimuli [35].

In the present study, we observed a potential link between ferroptosis and the comorbid migraine and painful TMDs [31]. By analyzing differential gene expressions using bulk RNA-seq, we identified 15 ferroptosis-related DEGs in the comorbidity condition. We further observed that ferroptosis-related genes are enriched in the pathways of neurodegeneration, cellular homeostasis, interleukin signaling, and pain response. These genes are implicated in various aspects of pain. For example, *Il1b* and *Lcn2* are key mediators of inflammatory and neurogenic pain [36], respectively, while *Nox1* and *Gpx2* are involved in oxidative stress, a critical factor in pain modulation [37]. The role of *Anxa2* in cellular stress and inflammation further highlights the link between ferroptosis and pain mechanisms [38]. *Vdr* and *Mir7-1* are implicated in inflammatory responses and neural signaling, respectively, to contribute to pain mechanisms [39,40]. The interplay between these genes suggests that targeting ferroptosis-related DEGs could develop novel therapies for the comorbidity of migraine and TMDs.

The connection between orofacial pain, ferroptosis, and immune responses involves complex mechanisms that contribute to the pathogenesis of pain conditions such as migraine and TMDs. Immune cells like macrophages, dendritic cells, neutrophils, and eosinophils infiltrate inflamed tissues and release pro-inflammatory cytokines and chemokines, thereby exacerbating pain. Notably, *Il1b*-encoded interleukin-1 beta (IL-1β) promotes inflammation and sensitizes nociceptors to heighten pain perception [41]. Consistent with *Il1b* emerging as a shared hub, a prior study using a familial hemiplegic migraine model reports IL1RN upregulation after cortical spreading depression [42]. This pattern suggests an endogenous brake on *Il1b*/IL1R1 signaling that we could exploit therapeutically. Mechanism-based options include direct IL-1 blockade with anakinra, rilonacept, or canakinumab, which show efficacy in IL-1–driven diseases such as Cryopyrin-Associated Periodic Syndromes and recurrent pericarditis [43,44]. In addition, upstream inhibition of the NLRP3 inflammasome reduces NTG-induced hyperalgesia in preclinical migraine models. First-generation agents have showed hepatotoxicity, but newer chemotypes, such as dapansutrile, show improved safety signals. Together, these data indicate the IL-1/NLRP3 axis as a testable therapeutic node in the comorbid migraine and TMDs [45,46,47]. Collectively, the network centrality of *Il1b*, its spatial enrichment in the Sp5C, its involvement in ferroptosis, and its potential druggability suggest the IL-1 axis as a mechanistic fulcrum in this comorbidity, which supports that prioritizing IL-1–directed interventions (e.g., IL-1 blockade or upstream NLRP3 inhibition) and/or targeting cell type–specific *Il1b* signaling could be developed into a novel therapy for such comorbid pain.

Ferroptosis, characterized by iron-dependent lipid peroxidation, leads to oxidative stress and cell death, which can activate immune cells and trigger inflammation. Genes like *Lcn2* and *Nox1* modulate iron homeostasis and ROS production, thereby impacting immune activity and pain pathways [48,49]. Oxidative stress from ferroptosis contributes to neuroinflammation and sensitizes neurons to enhance pain signaling. The release of damage-associated molecular patterns (DAMPs) from cell death further activates immune responses and sensitizes pain pathways [50]. The shared pathways, such as those regulated by *Il1b*, *Nox1*, and *Lcn2*, are involved in both ferroptosis and immune responses, suggesting that targeting these pathways could modulate multiple aspects of the comorbid migraine and TMDs. Inflammation can promote ferroptosis by increasing oxidative stress, while ferroptosis can enhance inflammation through ROS and DAMPs, which forms a feedback loop that exacerbates painful conditions [51].

Our study has the following limitations. First, bulk RNA-seq cannot resolve cell type-specific changes, and cutting-edge single-cell sequencing could better distinguish neuronal, glial, and immune contributions in the comorbidity condition. Second, the gene regulation revealed in this study is correlational, and we need to carry out further studies to determine the causal role of the identified hub genes in the comorbid orofacial pain. Third, public resources such as GTEx and the Human Protein Atlas do not provide expression data at the Sp5C level, thus we relied on region-matched quantification and curated mouse references for spatial context; we will incorporate Sp5C-resolved human datasets when they become available.

The interplay between orofacial pain, ferroptosis, and immune responses highlights the complexity of these conditions and the need for integrated therapeutic approaches. In the present study, we identified a set of ferroptosis-related genes that are differentially expressed in comorbid migraine and myogenic TMD in female mice. We also demonstrate that these DEGs map to pathways of immune signaling, oxidative stress, and neurodegeneration, and more importantly, IL1β/NLRP3 stands out as a convergent hub linking ferroptosis and inflammation. By targeting the shared pathways and mechanisms that link these processes, we can develop more effective treatments for managing the comorbidity of migraine and TMDs. Further research is essential to validate these connections and explore novel therapeutic strategies for this comorbidity condition.

## Data Availability

The original contributions presented in the study are included in the article/Supplementary Material, further inquiries can be directed to the corresponding author.

## Supplementary Materials

The following supporting information can be downloaded at: https://www.mdpi.com/article/10.3390/genes16121435/s1, Figure S1: Drug–gene interaction network of ferroptosis-related DEGs; Figure S2: Spatial expression patterns of selected hub genes in the mouse brainstem; Table S1: NTG vs. Sham; Table S2: TL vs. Sham; Table S3: TL-NTG vs. Sham.
