# Supplementary material for: Gene Regulation in Comorbid Migraine and Myogenic Temporomandibular Disorder Pain
Source: Genes (Basel). 2025 Dec 1;16(12):1435. doi: 10.3390/genes16121435 (PMC12733123; doi:10.3390/genes16121435)
Supplement: Supplementary file 1 [file genes-16-01435-s001.zip › Figure S1.pdf]

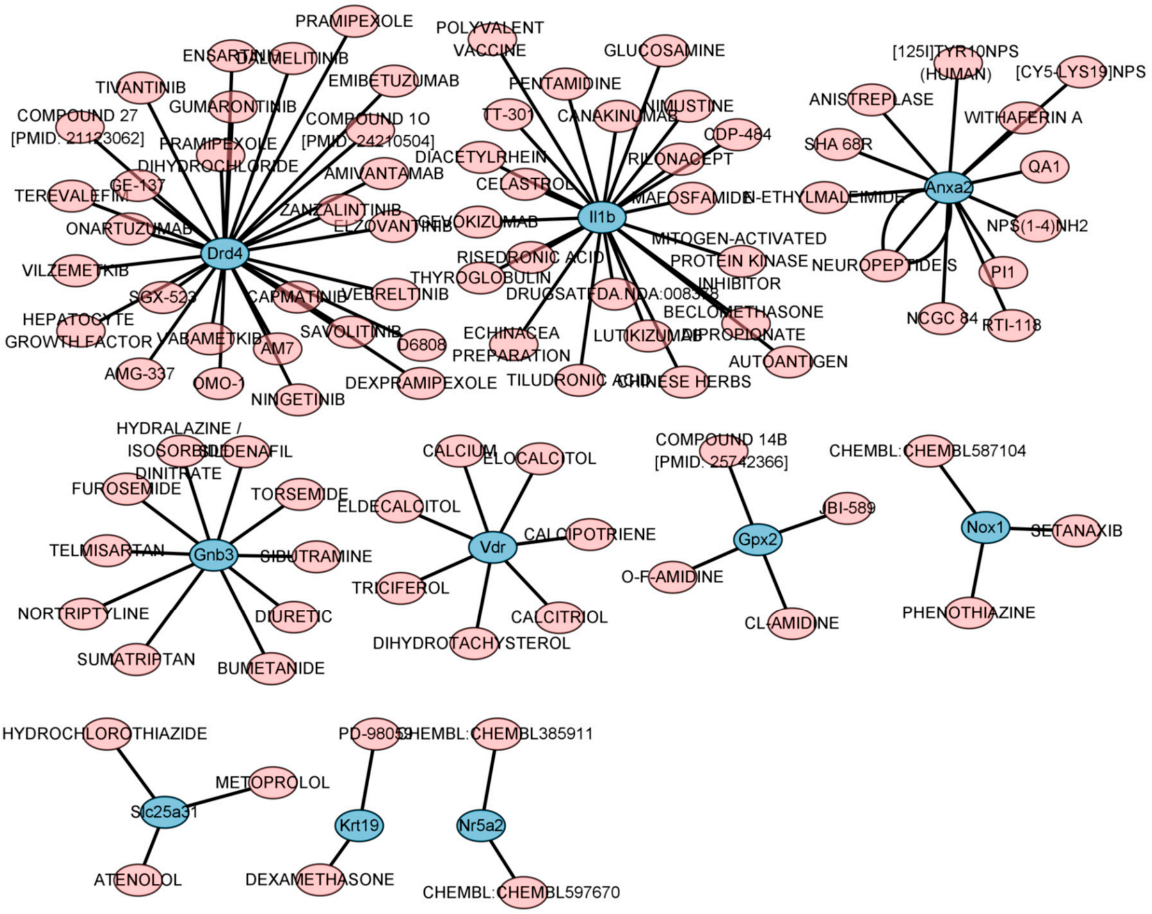

Figure S1. Drug–gene interaction network of ferroptosis-related DEGs. Ferroptosis-related DEGs were mapped to potential drug–gene interactions using the Drug–Gene Interaction Database (DGIdb). Blue nodes represent genes, and pink nodes represent drugs or compounds that target these genes. The network reveals multiple potential therapeutic agents interacting with the hub genes such as Il1b, Drd4, and Gna8.
