# Supplementary material for: Gene Regulation in Comorbid Migraine and Myogenic Temporomandibular Disorder Pain
Source: Genes (Basel). 2025 Dec 1;16(12):1435. doi: 10.3390/genes16121435 (PMC12733123; doi:10.3390/genes16121435)
Supplement: Supplementary file 1 [file genes-16-01435-s001.zip › Figure S2.pdf]

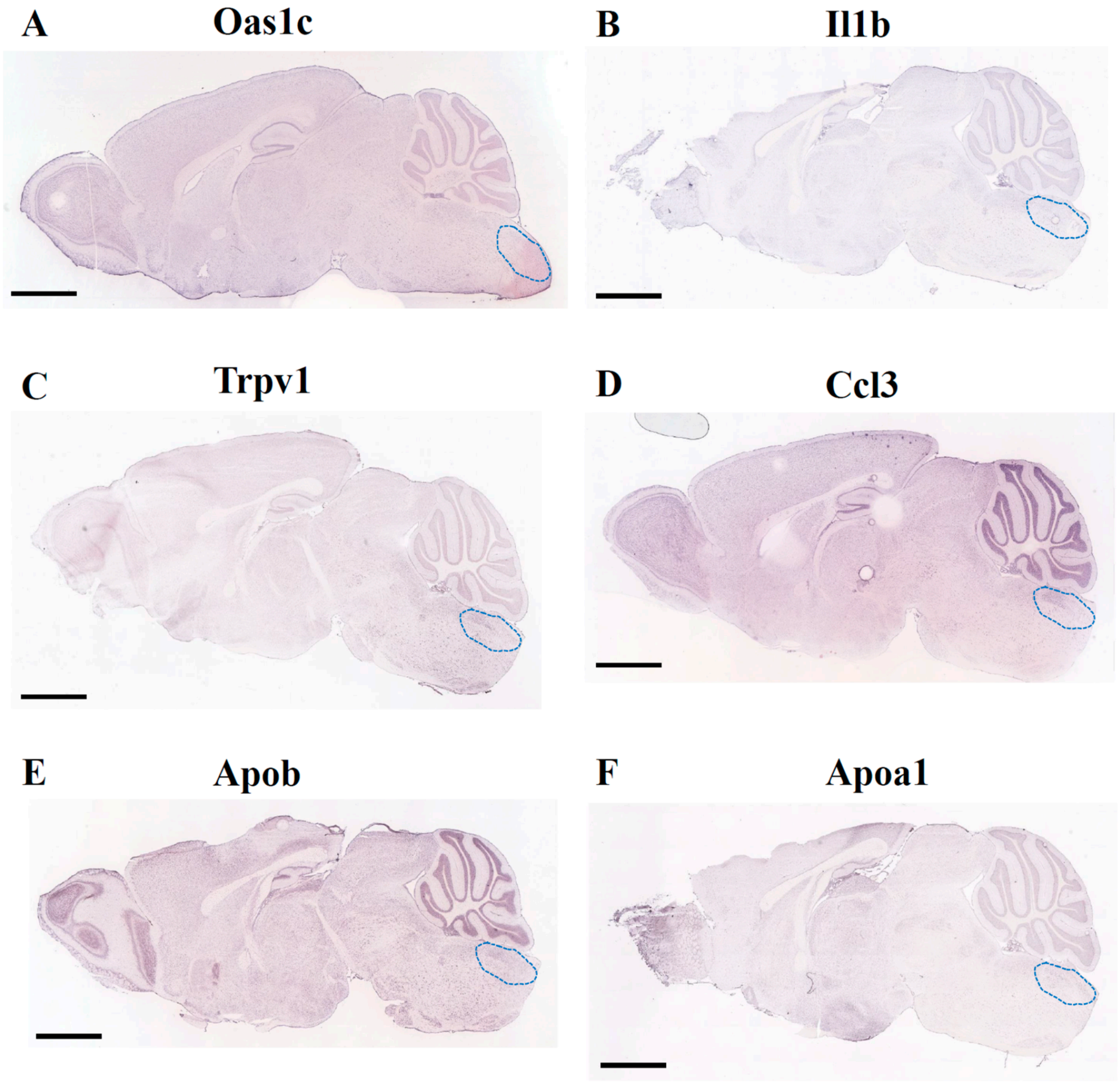

Figure S2. Spatial expression patterns of selected hub genes in the mouse brainstem. Representative sagittal brain sections showing in situ hybridization (ISH) signals for six hub genes: Oas1c (**A**), Il1b (**B**), Trpv1 (**C**), Ccl3 (**D**), Apob (**E**), and Apoa1 (**F**). The blue dashed circles highlight the Sp5C region. Expression intensity varies among genes, reflecting their differential localization within the brainstem. Scale bars = 1.3 mm.
